# Single-wall carbon nanotubes and graphene oxide-based saturable absorbers for low phase noise mode-locked fiber lasers

**DOI:** 10.1038/srep25266

**Published:** 2016-04-29

**Authors:** Xiaohui Li, Kan Wu, Zhipei Sun, Bo Meng, Yonggang Wang, Yishan Wang, Xuechao Yu, Xia Yu, Ying Zhang, Perry Ping Shum, Qi Jie Wang

**Affiliations:** 1 School of Physics and Information Technology, Shaanxi Normal University, Xi’an 710062, P.R. China; 2 Centre for Optoelectronics and Biophotonics, School of Electrical and Electronic Engineering, Nanyang Technological University, 50 Nanyang Ave., 639798, Singapore; 3 State Key Laboratory of Advanced Optical Communication Systems and Networks, Department of Electronic Engineering, Shanghai Jiao Tong University, Shanghai 200240, China; 4 Department of Micro- and Nanosciences, Aalto University, PO Box 13500, FI-00076 Aalto, Finland; 5 State Key Laboratory of Transient Optics and Photonics, Xi’an Institute of Optics and Precision Mechanics, Chinese Academy of Sciences, Xi’an 710119, China; 6 Singapore Institute of Manufacturing Technology, 71 Nanyang Drive, 638075 Singapore

## Abstract

Low phase noise mode-locked fiber laser finds important applications in telecommunication, ultrafast sciences, material science, and biology, etc. In this paper, two types of carbon nano-materials, i.e. single-wall carbon nanotube (SWNT) and graphene oxide (GO), are investigated as efficient saturable absorbers (SAs) to achieve low phase noise mode-locked fiber lasers. Various properties of these wall-paper SAs, such as saturable intensity, optical absorption and degree of purity, are found to be key factors determining the performance of the ultrafast pulses. Reduced-noise femtosecond fiber lasers based on such carbon-based SAs are experimentally demonstrated, for which the phase noise has been reduced by more than 10 dB for SWNT SAs and 8 dB for GO SAs at 10 kHz. To the best of our knowledge, this is the first investigation on the relationship between different carbon material based SAs and the phase noise of mode-locked lasers. This work paves the way to generate high-quality low phase noise ultrashort pulses in passively mode-locked fiber lasers.

Ultrashort optical pulses have been highly demanded in scientific studies and applications, e.g. high-speed optical laser sources for optical communication, optical frequency comb for optical metrology and time-resolved studies of ultrafast nonlinear phenomena, seed sources for laser amplifiers and supercontinuum sources, as well as the optical nano-machining for the materials processing etc^1^,^2^,^3^,^4^,^5^,^6^,^7^. Among different techniques, mode-locked laser technology is one of the most efficient means that generate ultrafast optical pulses. Low phase noise of mode-locked lasers is one of important devices in many real applications such as long-range and high-capacity optical fiber communications, high-resolution optical sampling, and frequency comb^8^,^9^,^10^. As a result, there are growing interests in developing low-phase noise ultrafast lasers.

Many saturable absorbers (SA) have been utilized to achieve mode locking, which can satisfy aforementioned requirements. Semiconductor saturable absorber mirrors (SESAMs) as one type of mode lockers are emerging as an enabling technology^11^. However, the fabrication typically requires complex and expensive manufacturing processes. Therefore, novel SAs with better characteristics (i.e. fast recovery time, suitable modulation depth, etc.), lower cost, and easier integration are of great interests^12^,^13^,^14^. Carbon materials have excellent performances, which can be potentially utilized as SAs for the generation of ultrashort pulses^15^,^16^,^17^. Single-wall carbon nanotube (SWNT) is a direct-bandgap material with its bandgap dependent on the diameter and chirality^15^,^18^. The fabricated nanotubes are often with hybrid chiralities, which lead to tremendous advantages in optoelectronics, such as large optical nonlinearity, environmental robustness, ultrafast carrier relaxation time (sub-picosecond), and low saturation intensity (SI)^12^,^13^,^19^,^20^,^21^,^22^,^23^,^24^,^25^. Graphene, as a two dimensional allotrope of carbon atoms in a honeycomb lattice with conical band structure has attracted great interests^26^, in broad photonic applications, such as broadband saturable absorbers (SAs)^27^,^28^,^29^,^30^,^31^, surface plasmonics, photodetectors^32^,^33^, optical modulator^34^, polarizers^35^, and transparent electrodes. Experiments also show that few-layered GO also has ultrafast carrier relaxation and large optical nonlinearities^36^,^37^,^38^, potential for ultrafast photonics^26^,^36^,^37^,^39^. All the carbon materials aforementioned have been demonstrated to have important applications in various ultrafast lasers^12^,^13^,^15^,^26^,^30^,^40^,^41^,^42^,, such as fiber^22^,^27^,^30^, waveguide^43^,^44^, solid-state^45^,^46^, and semiconductor lasers. In addition, these carbon materials exhibit various advantages, including but not limited to broadband absorption, large optical nonlinearity^12^,^13^, fast recovery time, easy to fabrication^47^, and low cost^48^.

The noise is mainly caused by the fluctuation of particular physical factors, such as the gain fluctuation, length and refractive index fluctuation, spontaneous emission fluctuations^49^. Carbon materials can be used to reduce the noise by introducing cavity loss to balance the gain and suppress spontaneous emission for a fixed fiber laser cavity. Controlling the noise characteristics (e.g., reducing timing jitter by 24%^50^, and reducing phase noise by 34%^51^.) by employing SWNT^52^ and graphene^51^ has been studied. These studies show that SWNT and graphene play key roles in reducing noise of generated pules^22^. However, various crucial factors of these SAs, such as operating wavelength, modulation depth (MD), and SI, which significantly influence the laser noise performance, have not yet been investigated. For a given passively mode-locked fiber lasers, the relationship of the phase noise with different SAs are still unknown at present.

Graphene based saturable absorbers have been studied intensively for phase noise reduction^51^. In order to broaden the knowledge of phase noise reduction about other two main carbon materials (i.e., SWNT and GO), we fabricate SWNT and GO SAs to study the phase noise performance in mode-locked fiber ring lasers. The fabrication processes of different materials have been optimized based on their different physic-chemical characteristics. Various methods (such as Raman spectra, linear absorption, and Scanning Electron Microscopy (SEM)) were utilized to characterize the fabrication process (e.g., purification of the SAs). The results show that the SWNT induced a higher loss than GO SA for each piece (PC). The GO SA shows fewer bubbles than SWNT SA, indicating that the GO SAs are purer than SWNT SAs. The phase noise performance of different wall-paper SAs with different number of PCs is compared in Erbium-doped fiber lasers, which operate in the anomalous dispersion regimes. Our experiments demonstrate that mode locking can be achieved by using different PCs of SA. The SWNT from 1 PC to 3 PCs can mode lock the fiber laser stably, so well as 1 PC to 4 PCs of the GO wall-paper SA. The results show that the phase noise can be optimized by varying the number of the PCs of SA films, through controlling the cavity loss and the saturable intensity of the absorbers. More than 10-dB phase noise can be reduced by controlling the parameter of SWNT SAs and more than 8-dB phase noise for GO SAs. The work demonstrated here could be applied in the design of various high-performance low-phase-noise mode-locked lasers.

## Results

### Sample preparation of the carbon material SAs

**Figure 1:**
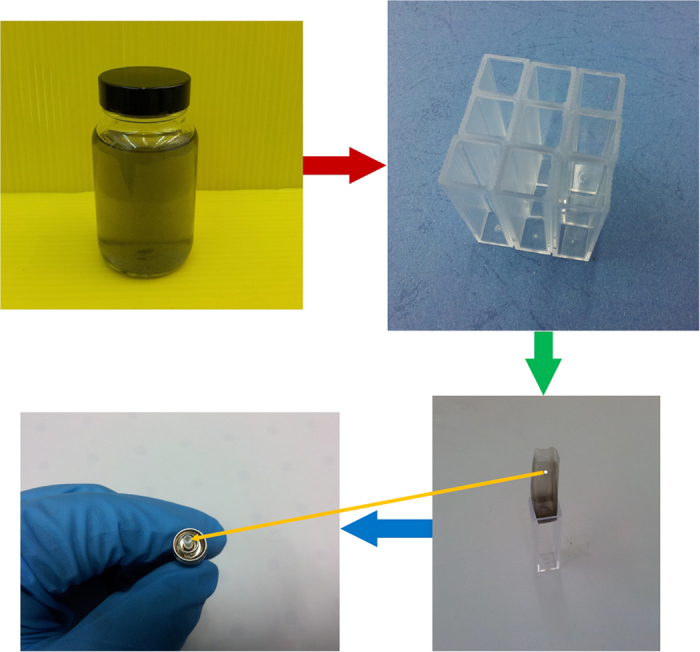
The fabrication processes for SWNT and GO-polyvinyl alcohol (PVA) SA components.

Carbon materials wall-paper absorbers (such as SWNT wall paper, GO wall paper as demonstrated here), fabricated by using vertical evaporation method, have been successfully applied in passively mode-locked lasers^42^,^53^. The vertical evaporation method and the mode locker preparing processes are shown in Fig. 1. The prepared SA solution is poured into the polystyrene cells. After evaporating for several days, The SA wall paper is formed in the cell, which can be easily stripped out of cell. We choose one small pure piece on the wall paper and attach it between the fiber connector. Detailed introduction on sample fabrication can be found in Supplementary document.

### Characterization of the carbon material SAs

We investigate different characteristics of the SWNT and GO SAs, which can potentially affect the performance of fiber lasers. Raman spectra, linear transmittance of the SA film, SEM of SWNT and GO wall papers, and nonlinear transmittance of different PCs of the SA films are studied.

**Figure 2:**
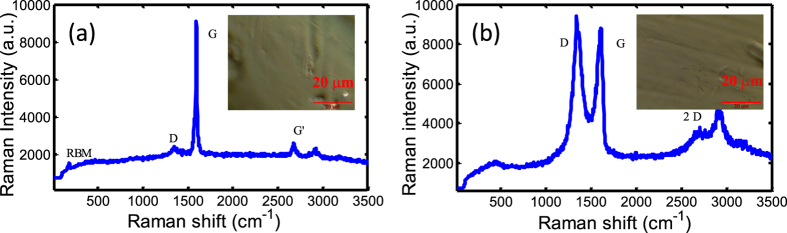
The Raman spectra of (**a**) SWNT-PVA (the inset shows micrograph of SWNT-PVA films) and (**b**) GO-PVA wall-paper SAs excited by a 532 nm laser (the inset shows micrograph of GO-PVA films).

The fabrication of different carbon materials mainly utilizes the vertical evaporation method^42^. However, different SAs have different processing ways due to their intrinsic characteristics. In order to characterize the SWNT- and GO-polyvinyl alcohol (PVA) wall-paper samples, Raman spectroscopy is utilized to measure the “finger-prints”. The spectrum of SWNT-PVA wall-paper SA is shown in Fig. 2(a). The inset is the micrograph of SWNT-PVA films. The SWNT sample is excited by a 532-nm laser. Different bands represent different characterization of SWNT wall papers. G-band (i.e. 1500–1600 cm^−1^) can describe the circumferential and axial vibrations. D band (i.e. 1300–1400 cm^−1^) is induced from the breathing motions of sp^2^ carbon atoms, which shows the defects of nanotube^54^. Radial-breathing-mode (RBM) band (100–400 cm^−1^), which can estimate the diameter of the tube, is a low frequency mode. It can be seen clearly from the Raman spectrum which is the characteristic of SWNT with high intensity of G-band. The background as seen from the Raman spectrum is due to the impurity, the optical scattering, and the PVA induced additional background noise. Figure 2(b) is the Raman spectrum of the GO-PVA films. The inset is the micrograph of GO-PVA films. There are some characteristic bands of D, G as seen from Raman spectrum. Different peaks represent different physical meanings. D band at around 1366 cm^−1^ shows the structural defects induced by attaching the hydroxyl and epoxide groups. G band at around 1610 cm^-1^ is related to the first-order E_2g_ mode^55^. Some peaks and substrate on the Raman spectrum are induced by polymeric materials^56^.

**Figure 3:**
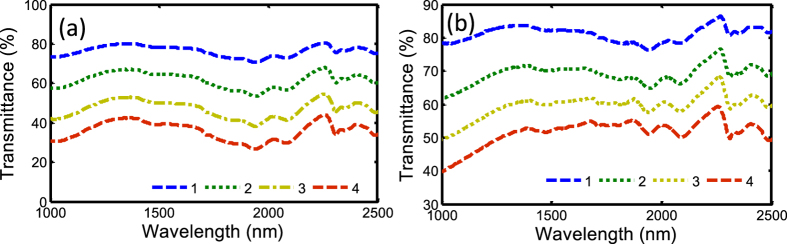
Linear transmittances of (**a**) SWNT-PVA and (**b**) GO-PVA wall-paper saturable absorbers with different number of PCs. The blue, green, yellow, and red dashed lines represent the 1, 2, 3, and 4 PCs of SAs, respectively.

An UV-Visible-NIR spectrophotometer (Agilent Technologies, Cary 5000) is used to measure the linear optical transmittance of the SWNT film with different PCs as shown in Fig. 3(a). The transmittance will decrease, with the increases the PCs of SAs. For example, the transmittance of single PC SWNT wall-paper absorber is 78.23%, while it decreases to 39.23% for four-PC SWNT absorber at 1550 nm, which induces much linear loss if it is inserted in the laser cavity PC by PC. The linear optical transmittance of different PCs of GO-PVA film is shown in Fig. 3(b). The transmittance of single PC GO absorber around 1550 nm is 82.13%. While increasing the PCs of the GO SAs, the transmittance becomes small. For a four-PC GO-PVA device, the transmittance reduces to 52.07%, which will cause about 3-dB loss in the fiber cavity. SWNT films induce slightly larger loss than GO films for the same number of PCs. This is because high SWNT concentration is used, which can be controlled during the fabrication process.

**Figure 4:**
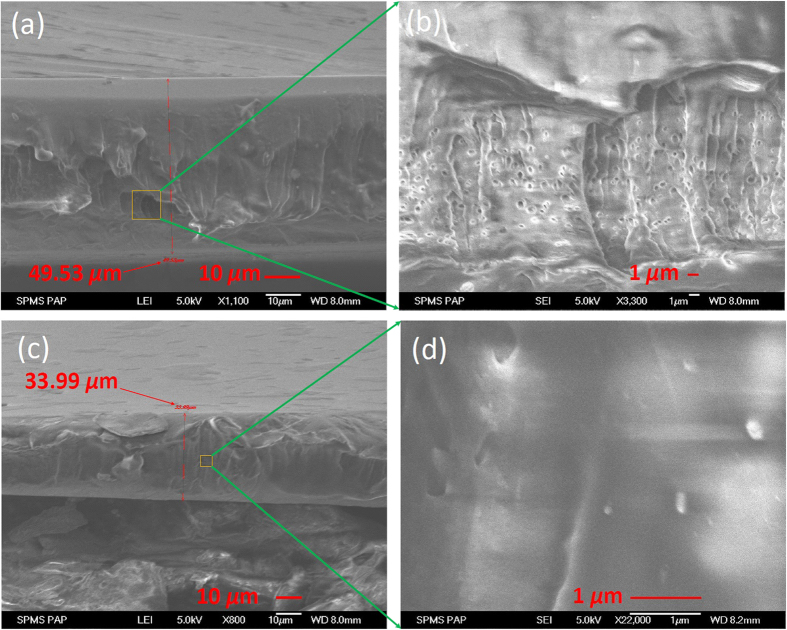
SEM images of the cross sections for (**a**) SWNT, (**c**) GO wall-papers SA in different scales and the corresponding zoom-in images of (**b**) SWNT, (**d**) GO SA.

We also check the uniformity of different SA polymer films (i.e., SWNT- and GO-PVA films). Figure 4(a,b) provide the scanning electron microscope (SEM) of cross-section for SWNT and GO thin films. The average thickness of the SWNT SA is about 49.53 μm as show in Fig. 4(a). The SWNT-PVA is not pure at one side of the surface. Figure 4(b) shows the zoom-in image of the SWNT SA in a smaller range. We can see there are some small bubbles and large holes which significantly increase the impurity of the SAs. As a result, certain optical scattering effects lead to loss in the fiber cavity when it used as SAs. Figure 4(c,d) show the SEM of the GO wall-paper absorbers in different ranges. The average thickness of GO wall-paper absorber is 33.99 μm as shown in Fig. 4(c). The roughness of the facet is due to the cutting tool of the scissors. GO can be dispersed very well into water, so the fabrication processing is free of any surfactants such as sodium dodecyl sulfate (SDS), which can significantly reduce the non-saturable scattering losses of the SAs. As shown in Fig. 4(d), there are less air holes or bubbles in the GO-PVA polymer film, which shows the improved uniformity of the SA. The bright side is due to the less free electrons in this region during the measurement.

The nonlinear saturable absorption property of SWNT and GO SAs can be expressed as^57^


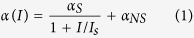


where *I*_*S*_ is the SI, 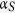 and 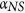 are saturable and nonsaturable absorbance, respectively.

**Figure 5:**
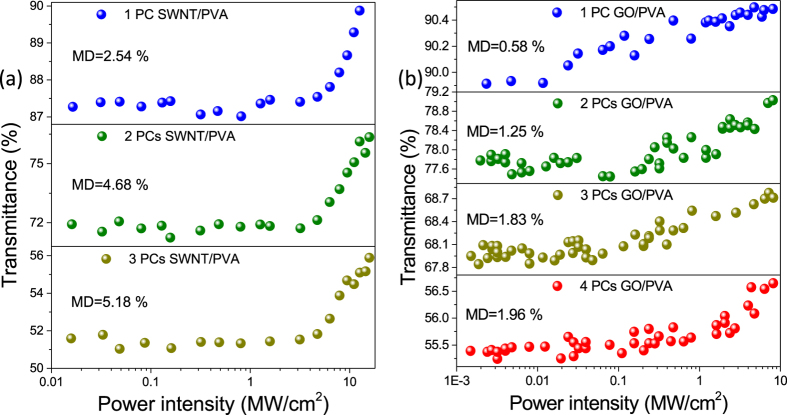
Nonlinear transmittances with different pieces of (**a**) SWNT- and (**b**) GO-PVA SAs. The measurement wavelength is 1560 nm.

Since MD and SI are very important for the pulse shaping and the noise properties, we investigate the nonlinear transmittance of different SA films with different PCs. The nonlinear transmittance of the SA with different PCs is measured based on power-dependent measurements^57^,^58^. We use a commercial Er-doped fs fiber laser (MENDOCINO Femtosecond Fiber laser, CALMAR LASER Company) as a laser source, which has pulse width of 80 fs, repetition rate of 50 MHz, and maximum average output power of 80 mW. The input power to the SA films is controlled by means of a variable optical attenuator. A reference signal is used to detect the incident power with an OC. Different PCs of the SWNT- and GO-PVA thin films, cut into small (2 mm^2^) composites, are directly sandwiched between fiber connectors. The performance of various SWNT- and GO-PVA SAs is shown in Fig. 5(a,b), respectively. The MD for 1, 2, and 3 PCs of SWNT-PVA wall paper are 2.54%, 4.68%, and 5.18%, respectively. 3-PC SWNT-PVA SA will induce almost 44.34% nonsaturable absorption loss in the fiber-based SA components. The MD of 1, 2, 3, and 4 PCs of GO-PVA wall paper are 0.58%, 1.25%, 1.83%, and 1.96%, respectively.

The measured nonlinear properties are summarized in Table 1. We can see that the single-PC SWNT-PVA and GO-PVA SA components have the SI of 9.00 and 0.205 MW/cm^2^. With the increase of the number of PCs, the nonsaturable losses are increased linearly for the two sets of SAs. However, the saturation intensities decrease but not strictly linear decrease. The thickness of the SA films plays the predominant role for the nonsaturable losses. We attribute the variation of saturation intensities to the non-uniform of the SA films and the optical scattering effect. The MDs are also varied, with different number of the PCs. In general, SWNT-PVA SA fiber components have larger MD than GO-PVA SA fiber components, while the SI of the GO-PVA SA is lower than the one of SWNT-PVA SA. When we increase the input power that the power intensity reaches 10 MW/cm^2^, it may near the damage threshold of the PVA. As a result, the SI will become low.

### Experimental setup

**Figure 6:**
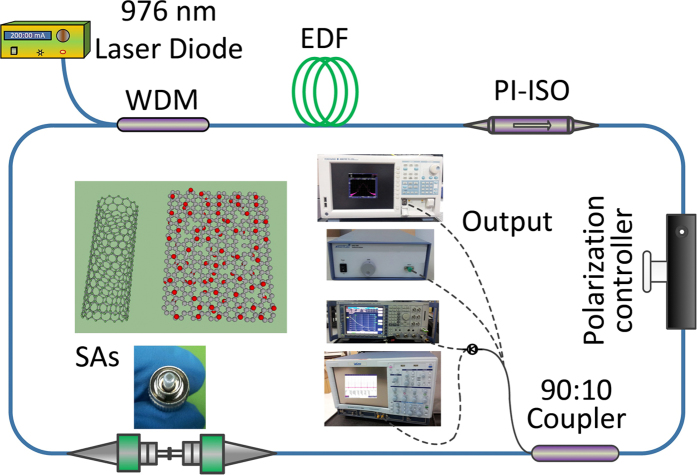
Experimental setup of the ultrafast-laser based on SWNT- or GO-PVA SAs.

In the experiments, we investigate the carbon materials SAs affecting on the performance of mode-locked fiber ring laser. The experimental setup with different SAs is designed as shown in Fig. 6. The fiber ring laser mainly consists of an Erbium-doped fiber (EDF) with length of about 0.8 m, some fiber components, and different sections of single-mode fibers. In order to prevent the laser reflecting and ensure that mode locking can be obtained solely by SAs, An in-line polarization insensitive isolator (PI-ISO) is spliced with the active fiber. One polarization controller is utilized to control the weak birefringence of the fiber ring cavity to assistant the mode locking. The 976-nm pump laser is coupled into the fiber ring resonator with one 980/1550 wavelength division multiplexing (WDM) coupler. The pulse can be coupled out of the ring cavity through a 10:90 output coupler (OC) with 10% port. Two kinds of SAs films (SWNT-, GO-PVA films) are inserted between fiber connectors. The output performances are measured by different commercial equipment. A signal source analyzer (Rohde & Schwarz FSUP26) together with a 2-GHz photodetector is used to measure the output of mode-locked pulse train. In this work, we use the von der Linde’s method^59^ to measure the result. The spectral power density (PSD) of mode-locked pulse train can be measured by a high speed photodetector, which represents the quantum noise. An autocorrelator and an optical spectral analyzer (OSA) are used to measure the pulse duration and output spectrum, respectively.

## Experimental Results

The outputs are recorded when the fiber laser is mode locked with fundamental repetition rate at the maximum pump strength without spurs as seen from the spectra. The PCs of different SAs between the fiber connector can be varied to demonstrate the relationship between the thickness of the SAs and the laser operation. The experimental results show in Table 2.

**Figure 7:**
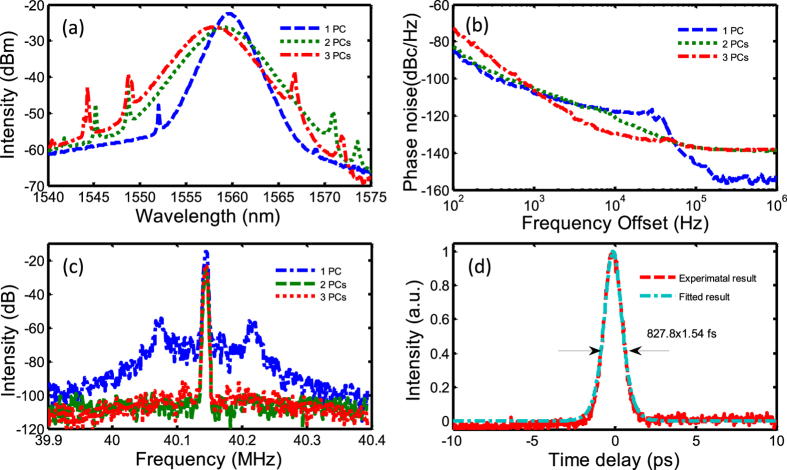
Experimental results with SWNT wall-paper saturable absorbers. (**a**) Mode-locked optical spectra of SWNT SAs with 1~3 PCs, corresponding (**b**) phase noise spectra, (**c**) RF spectra, (**d**) autocorrelation trace of the optical pulse for the mode locking with 3-PC SWNT SA.

SWNT wall papers from 1 to 3 PCs are used in the fiber laser cavity to achieve mode locking successfully. Detailed experimental results based on SWNT wall paper are shown in Fig. 7. Some output performances, such as optical spectra, phase noise, RF spectra for different PCs SWNT wall paper, and the pulse width for 3-PC SWNT absorber are shown in Fig. 7(a–d), respectively. The spectral widths increase from 2.51 to 4.53 nm, when the mode locking can be obtained from 1 to 3 PCs. However, the output power decreases from 551 μW to 395 μW, which indicates the increasing loss induced in fiber cavity with the increase of the number of the SA PCs. The pulse width of mode-locking using 3-PC SWNT SAs is about 827.8 fs (sech^2^-profile is assumed).

It can be observed that relaxation oscillation exists in the RF spectrum and phase noise spectrum for the laser mode locked with 1-PC SWNT SA. Relaxation oscillation leads to the perturbation of the mode locking operation and thus is not desired. Increasing the MD is expected to suppress it, which is confirmed when 2-PC and 3-PC SWNT SA are incorporated in the laser cavity. For the 2-PC SWNT SA, very weak relaxation oscillation still exists as seen from the phase noise spectrum. For the 3-PC SWNT SA, the relaxation oscillation is completely suppressed and leads to much low phase noise from 1 kHz to 50 kHz offset frequency. A phase noise reduction of 8.6 dB is observed at 10 kHz compared with 2-PC SWNT SA based mode locking and 11.5 dB compared with 1-PC SWNT SA based mode locking. However, due to the higher non-saturable loss in the SA, the laser has even lower output power and the phase noise at low offset frequency (below 1 kHz) becomes higher. Phase noise is increased by ~8 dB at 100 Hz.

**Figure 8:**
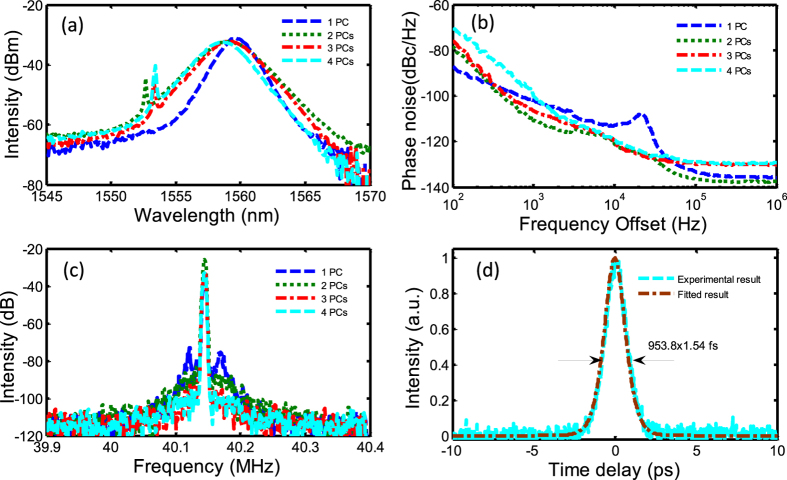
Experimental results with GO wall-paper saturable absorbers. (**a**) Mode-locked optical spectra of GO SAs with 1~4 PCs, (**b**) corresponding phase noise spectra, (**c**) RF spectra, (**d**) autocorrelation trace of the optical pulse for the mode locking with 4-PC of GO SA.

GO wall paper can mode lock the fiber laser from 1 to 4 PCs. Mode-locked spectra, phase noises, and corresponding RF spectra for different PCs can be obtained from Fig. 8(a–c), respectively. The autocorrelation trace of mode locking with 4-PC GO absorber is shown in Fig. 8(d). If a sech^2^-profile is assumed, the pulse width is about 953.8 fs. The spectral width varies from 1.6 nm to 2.9 nm and the output power varies from 299 μW to 212 μW with the increase of the PCs of GO SAs from 1 to 4.

It can be observed that the relaxation oscillation also exists for the laser mode locked with 1-PC GO SA. Relaxation oscillation is suppressed when 2-PC GO SA is incorporated in the laser cavity which results in a reduction in the whole measured phase noise spectrum. Phase noise reductions of 8.8 dB at 1 kHz and 7.6 dB at 10 kHz are obtained compared with the laser mode locked by 1-PC GO SA. Because the higher non-saturable loss reduces the laser output power and pulse energy, further increasing the number of GO SA PCs leads to the increase of the phase noise. The laser cannot be mode locked when more than 4 PCs of GO SA are incorporated in the cavity.

The optimization of the phase noise is a complex balance between different laser parameters. For the SA-PVA thin film fabricated here, a moderate MD (i.e., a proper number of SA PCs) helps to suppress the relaxation oscillation which represents the instability of the mode locking. Meanwhile too strong MD introduces both higher saturable loss and non-saturable loss which increases the cavity loss, reduces the pulse energy and bandwidth and finally leads to the worsening of the laser phase noise.

It can also be seen that SWNT SA and GO SAs exhibit different mode locking and noise properties in the laser^60^. This indicates that MD and SI also play important roles in the laser operation. Higher SI in SWNT SA results in higher mode locking threshold and thus higher pulse energy and wider optical spectrum, which should improve the phase noise. However, higher saturable loss and non-saturable loss partially counteract the benefits. As a result, the phase noises in the SWNT SA based laser and GO SA based laser are comparable to each other. By improving the fabrication process and reducing the nonsaturable loss, the SA may further help to optimize the phase noise performance of the mode-locked lasers.

There are also some parameters such as the dispersion, the nonlinearity, and even the operation wavelength, except the saturable absorber itself^61^. All the parameters need to be optimized in order to get the lowest phase noise. In addition, the mechanism of different SAs, such as nonlinear polarization evolution, SESAM, and carbon materials, are quite different. It can’t be directly pointed out which one is better for the noise reductions.

In this work, we propose a way to reduce the phase noise a lot solely by changing the characteristic of the saturable absorbers, such as the thickness, MD, and the linear absorptions. After optimization of all the parameters above, the phase noise could be further improved.

In summary, the method to achieve high-quality ultrashort pulses in the mode-locked lasers is investigated. Different carbon materials (SWNT, GO) as SAs are evaluated systematically for the application in low-noise passively mode-locked lasers. The characteristics of the wall-paper SAs, such as transmittance, SI, MD, and degree of purity, can be controlled by changing the number of the SA pieces and the fabrication method. It shows that the number of SA pieces influences the noise characteristics of mode locking. By optimizing the number of SA pieces, relaxation oscillation can be completely suppressed and the phase noise can be reduced by ~10 dB at 10 kHz in a laser mode locked by SWNT-PVA SA and more than 8 dB by GO-PVA in the experiments. This work proposes a convenient method to adjust the SA parameters and achieve high quality ultrashort pulses from the mode-locked lasers. Moreover, the method is not limited to SWNT and GO but can be applied to any SA embedded in the thin film structure.

## Additional Information

**How to cite this article**: Li, X. *et al.* Single-wall carbon nanotubes and graphene oxide-based saturable absorbers for low phase noise mode-locked fiber lasers. *Sci. Rep.* **6**, 25266; doi: 10.1038/srep25266 (2016).

## Supplementary Material

Supplementary Information

## Figures and Tables

**Table 1:** Nonlinear optical properties of SWNT- and GO-PVA integrated fiber devices.

SAs	Numbers of PCs	Transmission		Nonlinear transmission parameters		Saturation intensity(MW/cm^2^)
		Starting point (%)	Saturation (%)	Nonsaturable loss (%)	Modulation depth (%)	
SWNT-PVA SAs	1	87.38	89.92	10.08	2.54	9.00
	2	71.67	76.35	23.65	4.68	9.19
	3	50.48	55.66	44.34	5.18	9.66
GO-PVA SAs	1	89.91	90.49	9.51	0.58	0.205
	2	77.78	79.03	20.97	1.25	1.786
	3	67.95	68.78	31.22	1.83	1.820
	4	55.39	56.65	43.35	1.96	2.254

**Table 2:** Output performances of the mode-locked fiber ring lasers with SWNT- and GO-PVA SA.

SAs	Numbers of PCs	Pump current (mA)	Output power (μW)	Spectral bandwidth (nm)	Center wavelength (nm)	RF carrier power (dBm)	Phase noise@ 1 kHz (dBc/Hz)	Phase noise@1 kHz (dBc/Hz)	Relaxation oscillation
SWNT-PVA	1	207	551	2.51	1559	−19.75	−107.7	−117.8	Yes
	2	234	438	4.5	1559	−25.56	−105.2	−120.7	No
	3	293	395	4.53	1558	−23	−106	−129.3	No
GO-PVA	1	156	299	1.6	1559	−28.1	−102	−113.1	Yes
	2	168	212	2.26	1558.9	−26.2	−110.8	−120.7	No
	3	184	277	2.9	1559	−32.5	−106.6	−120.6	No
	4	203	289.7	2.79	1558	−33	−100	−119.4	No
