# Supplementary material for: Single-wall carbon nanotubes and graphene oxide-based saturable absorbers for low phase noise mode-locked fiber lasers
Source: Sci Rep. 2016 Apr 29;6:25266. doi: 10.1038/srep25266 (PMC4850480; doi:10.1038/srep25266)
Supplement: Supplementary Information [file srep25266-s1.pdf]

Single-walled carbon nanotubes and graphene oxide-based saturable absorbers for low phase noise mode-locked fiber lasers (Supplementary)

Xiaohui Li,[1, 6‡] Kan Wu,[2‡] Zhipei Sun,[3] Bo Meng,[1] Yonggang Wang,[4] Yishan Wang,[4] Xuechao Yu,[1] Xia Yu,[5, **] Ying Zhang,[5] Perry Ping Shum,[1] Qi Jie Wang[1, *]

1. OPTIMUS, Centre for Optoelectronics and Biophotonics, School of Electrical and Electronic Engineering, Nanyang Technological University, 50 Nanyang Ave., 639798, Singapore

2. State Key Laboratory of Advanced Optical Communication Systems and Networks, Department of Electronic Engineering, Shanghai Jiao Tong University, Shanghai 200240, China

3. Department of Micro- and Nanosciences, Aalto University, PO Box 13500, FI-00076 Aalto, Finland

4. State Key Laboratory of Transient Optics and Photonics, Xi'an Institute of Optics and Precision Mechanics, Chinese Academy of Sciences, Xi'an 710119, China

5. Singapore Institute of Manufacturing Technology, 71 Nanyang Drive, 638075 Singapore

6. School of Physics and Information Technology, Shaanxi Normal University, Xi'an 710062, P.R. China.

Correspondence and requests for materials should be addressed to Q. J. W. (*qjwang@ntu.edu.sg) X. Y. (**xyu@SIMTech.a-star.edu.sg)

1. SWNT wall paper preparation.

The SWNTs used in the experiments were grown by electric arc discharge technique. The mean diameter of the SWNTs is about 1.5 nm. Since different diameter of the SWNT is corresponding to different mode-locked wavelength. The vertical evaporation method is used to preparing the SWNT wall paper SAs. We have made many SAs with different density (the amount of SWNT in the wall paper SAs). In this experiment, 0.6 mg of the SWNT powder were poured into 10 ml 0.1% sodium dodecyl sulfate (SDS) aqueous solution, which act as a surfactant. After the ultrasonic process of the SWNT aqueous solution for 10 hours, the dispersed solution of SWNT was centrifuged to induce sedimentation of large SWNT bundles. After decanting the upper portion of the centrifuged solution, some PVA powder was poured into the solution and dissolved at 90 ºC with ultrasonic agitation for 3 hours. Then, the SWNT-PVA dispersion was diluted and poured into a polystyrene cell. Finally, we put these cells in an oven for evaporation. When the evaporation was finished, the wall and the bottom of the cell were coated with a thin plastic film. The PVA aqueous solution has strong viscosity to the polystyrene cell so that it adheres to the wall of the cell. When the cell was dry, the PVA film lost the viscosity to the cell, so we can strip the PVA film cell off the polystyrene cell by a tweezers easily. Due to the gravity effect, most of the materials are at the bottom with the thickness of more than 5 mm. The thickness of film on the wall of cell is far smaller than the one at the bottom. It is true that the film on the wall is suitable to insert between fiber connecters to let the light propagating through the film and neglecting the thickness itself. In the experiment, we cut the wall paper SA into small square shape pieces and insert it between the fiber connecters to make a sandwich structures.

2. Graphene oxide wall paper preparation.

The GO sheets used in this experiment were fabricated by ultrasonic agitation after chemical oxidation of graphite. There are some different between fabrication of GO-PVA absorber and SWNT-PVA. Generally, SWNT/polymer composite absorbers must contain surfactant impurity in the fabrication of SWNT dispersion. The impurity may increase the non-saturable losses and strong scattering in the composite film. GO can be dispersed very well into water without surfactant such as SDS, thus reducing the non-saturable scattering losses related to surfactant impurity.

The GO dispersion and GO-PVA solution were fabricated without surfactant so as to decrease impurities in the absorber. The characteristics of the GO-PVA film can be controlled by modulating the composition of the GO and PVA in the aqueous solution. The increase of the composition of the GO in the solution will lead to the increase of saturable absorption and

non-saturable losses, while enhancing the content of PVA in the solution will increase the hardness of the film. Therefore, both GO and PVA should be optimized to improve the mechanical and optical properties of the absorber. In this experiment, the 2.5 mg GO powder powders together with 0.6 g PVA were used to form a SA. Due to the strong viscosity of the PVA aqueous solution, the GO-PVA composite adheres to the wall of the polystyrene cuvette cell. When the evaporation was finished, the GO-PVA film lost the viscosity so that it can be stripped off the polystyrene cell easily by a pair of tweezers. We choose one small pure piece on the wall paper and attach it between the fiber connector.
